# Handgrip Strength and Phase Angle Predict Outcome After Bariatric Surgery

**DOI:** 10.1007/s11695-020-04869-7

**Published:** 2020-08-15

**Authors:** A. L. H. Gerken, K-K. Rohr-Kräutle, C. Weiss, S. Seyfried, C. Reissfelder, G. Vassilev, M. Otto

**Affiliations:** 1 Department of Surgery, Medical Faculty Mannheim, Universitätsmedizin Mannheim, Heidelberg University, Theodor-Kutzer-Ufer 1-3, 68167 Mannheim, Germany; 2 Department of Biometry and Statistics, Medical Faculty Mannheim, Universitätsmedizin Mannheim, Heidelberg University, Theodor-Kutzer-Ufer 1-3, 68167 Mannheim, Germany

**Keywords:** Phase angle, Static muscle strength, Roux-en-Y gastric bypass, Sleeve gastrectomy, Body composition, Bioelectrical impedance analysis, BIA, Weight reduction

## Abstract

**Background:**

The amount of postoperative weight loss after bariatric surgery varies interindividually. The quality of the pre- and postoperative body composition is an important predictor of success. The aim of this study was to investigate the role of preoperative handgrip strength and phase angle (PhA) as predictors of sustained postoperative weight loss in order to assess the influence of body composition on the postoperative outcome after bariatric surgery.

**Method:**

In a prospective cohort study, bioelectrical impedance and follow-up data of 198 patients after laparoscopic sleeve gastrectomy (SG; *n* = 68) and Roux-en-Y gastric bypass (GB; *n* = 130) were analyzed for a period of 36 months postoperatively.

**Results:**

The mean preoperative handgrip strength (31.48 kg, SD 9.97) correlates significantly with the postoperative body composition up to 24 months after surgery. Preoperative PhA, gender, size, and body weight influenced postoperative weight loss significantly. A significant correlation between preoperative PhA (mean 6.18°, SD 0.89°) and total weight loss (%TWL) was observed up to 3 months after SG (*r* = 0.31444, *p* = 0.0218) and up to 12 months after GB (*r* = 0.19184, *p* = 0.0467). The optimum cutoff for the prediction of a response of less than 50% excess weight loss was a preoperative PhA of 6.0°.

**Conclusions:**

The preoperative handgrip strength confirmed its suitability for use as a predictor of postoperative body composition, whereas the preoperative PhA predicts postoperative weight loss after bariatric surgery. Further research is necessary to identify the role of these parameters for preconditioning.

**Electronic supplementary material:**

The online version of this article (10.1007/s11695-020-04869-7) contains supplementary material, which is available to authorized users.

## Introduction

Obesity is associated with severe comorbidities leading to increased mortality. Bariatric surgery is an effective therapy decreasing overall mortality [1]. Laparoscopic sleeve gastrectomy (SG) and laparoscopic Roux-en-Y gastric bypass (GB) are the most commonly performed procedures for effective and sustained weight loss.

The remission of comorbidities associated with obesity is a means for ascertaining the success of bariatric surgery [2, 3]. The primary aim of bariatric surgery, however, is weight loss. The effectiveness of weight loss varies between individual patients.

The preoperative identification of patients who potentially will not lose sufficient weight after a bariatric procedure is important for patient selection and offers the opportunity for preoperative interventions to improve the postoperative outcome. Potentially modifiable factors reflecting the patients’ muscular status and the quality of body composition, namely, preoperative handgrip strength and phase angle (PhA), have been shown to be able to predict postoperative weight loss [4, 5].

Bioelectrical impedance analysis (BIA) is commonly performed for the evaluation of pre- and postoperative body composition delivering the parameters body cell mass (BCM), extracellular mass (ECM), lean body mass (LBM), and body fat. The PhA reflects the quality of LBM. Mathematically, it represents the angular transformation of the phase shift of capacitance behind the voltage caused by body component resistance when applying a current. A low PhA is caused by decreased cell integrity.

The aim of this study was to validate the role of preoperative PhA and handgrip strength as predictors of postbariatric success in terms of sustained weight loss and postoperative body composition. The preoperative PhA has been proven to be associated with postoperative weight loss. In a small series, preoperative handgrip strength exhibited a predictive ability for postoperative body composition but neglecting the potential additional effect of PhA. In contrast to these previous studies and in order to validate the previous findings, we regarded both PhA and handgrip strength to determine their correlation with postoperative body composition and the amount of weight loss after bariatric surgery in a larger and different patient cohort with a longer follow-up.

## Materials and Methods

### Subjects

All patients who underwent a laparoscopic SG or laparoscopic Roux-en-Y GB at the University Medical Center Mannheim between January 2013 and December 2016 were included prospectively in this study. PhA and handgrip strength were recorded before the operation. Patients were excluded from this study whose measurements for preoperative handgrip strength, BIA, or PhA were missing; who lacked follow-up BIA measurements; who had undergone relevant cointerventions; whose follow-up was incomplete (lost to follow-up 6 months after surgery); or who died postoperatively.

Approval from the local institutional review board at the University Medical Center Mannheim was obtained, and the study was performed in accordance with the Declaration of Helsinki.

### Bioelectrical Impedance Analysis

BIA measurements were performed before and after the operation. Baseline body measurements were taken preoperatively after a 2-week period on a liquid low-calorie diet. Body weight and body composition were assessed by BIA as published previously by Otto et al. [6] and Vassilev et al. [5]. We assigned follow-up dates for postoperative assessment to certain follow-up periods (Supplementary Material).

### Static Muscle Strength

The handgrip strength of the dominant and nondominant hand was measured as published elsewhere [6]. The measurements were repeated three times. The mean value of the dominant hand was calculated and used for this study.

### Phase Angle

The PhA was measured preoperatively as published earlier [5].

### Statistical Analysis

All statistical calculations were performed using the SAS software, release 9.4 (SAS Institute Inc., Cary, NC, USA). For qualitative factors, absolute and relative frequencies are presented. Quantitative variables are described by their mean value together with standard deviation. In order to compare the two treatment groups with regard to a binary factor (e.g., patient’s sex), a chi-square test was used. Two mean values were compared with a two-sample *t* test if data were approximately normally distributed. For skewed data, the Mann-Whitney *U* test was used instead.

For repeated measurements, ANOVA was performed in order to investigate changes over time using the SAS procedure PROC MIXED (with the patient’s ID as a random factor and the measurement time as a fixed factor). In order to compare the treatment groups at a certain time point regarding a quantitative variable, an analysis of covariance adjusted for the baseline parameter was applied.

Univariable logistic regression analysis was performed for testing the influence of preoperative PhA on the binary outcome, determining “less pronounced” or “pronounced” weight loss after bariatric surgery, separately for SG and for GB. An excess weight loss (%EWL) result of less than 50% 12 months after surgery was defined as a “less pronounced weight loss” after bariatric surgery. In the case of missing values (e.g., patients lost to follow-up) 12 months postoperatively, we attested pronounced weight reduction after surgery to those patients with %EWL more than 50% 4–9 months after surgery.

Furthermore, in order to test the impact of several factors on the success of bariatric surgery simultaneously and to control for confounders, a multivariable logistic regression analysis was performed including all variables possibly leading to a response of less than 50% EWL, e.g., preoperative BMI. This analysis was performed using the “selection = forward” option of the SAS procedure PROC LOGISTIC. A receiver operating characteristic (ROC) curve was generated for each predictive variable.

In general, the result of a statistical test was considered significant for *p* less than 0.05. Only for the multiple logistic regression analysis was a significance level of alpha = 0.10 assumed.

## Results

A total of 198 patients, who underwent laparoscopic SG and GB, were included in this prospective cohort analysis. The number of patient exclusion together with reasons and the completeness of follow-up are reported in the Supplementary Material.

### Baseline Characteristics

Table 1 shows that the treatment groups differed markedly in their baseline characteristics regarding BMI. The preoperative body weight and BMI were higher in the SG group whereas the proportion of female patients was lower in the SG group. Therefore, it seemed to be necessary to adjust for baseline characteristics, e.g., for preoperative BMI, when comparing the treatment groups postoperatively. The preoperative handgrip strength and the PhA did not differ significantly between the SG and GB groups (Table 1).

**Table 1:** Characteristics of the patients in the study

	SG	GB	*p* value
*n* (%)	68 (34.3%)	130 (65.7%)	
Age (mean ± SD)	41.21 ± 12.06	42.42 ± 11.99	*p* = 0.4991
Gender male (*n*, %)	26 (38.2%)	23 (17.7%)	*p = 0.0015*
BMI (kg/m^2^) (mean ± SD)	54.28 ± 8.22	45.93 ± 5.24	*p < 0.0001*
Body weight (kg) (mean ± SD)	158.98 ± 28.41	128.69 ± 17.78	*p < 0.0001*
Handgrip strength* (kg) (mean ± SD)	33.29 ± 11.94	30.54 ± 8.66	*p* = 0.2759
PhA (°) (mean ± SD)	6.30 ± 1.09	6.11 ± 0.77	*p* = 0.5714

*BMI* body mass index, *BP* Roux-en-Y gastric bypass, *PhA* phase angle, *SG* sleeve gastrectomy

*Dominant hand

### Postoperative Changes over Time

The postoperative course of weight loss, as well as mean phase angle, body mass index, and bioelectrical impedance analysis, is presented as supplementary material (Supplementary Tables 1 and 2). There was no significant difference regarding the outcomes after SG compared with GB when adjusted for baseline values.

### Correlation Analyses

#### Correlation Between Preoperative Handgrip Strength and Preoperative Phase Angle

There was a significant correlation between the preoperative handgrip strength of the dominant hand and the preoperative PhA (*r* = 0.22506, *p* = 0.0014).

##### Correlation of Phase Angle with Weight Loss and Postoperative Body Composition

**Fig. 1:**
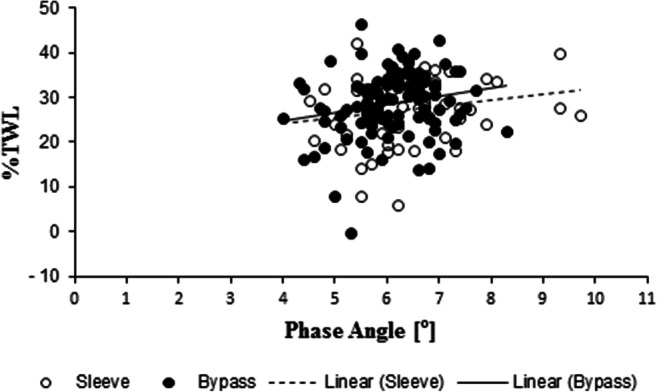
Correlation between the preoperative phase angle and the percentage of total weight loss (%TWL) 12 months after sleeve gastrectomy (SG) and gastric bypass (GB). Pearson’s correlation coefficients are *r* = 0.19988 (*p* = 0.1360) for SG and *r* = 0.19184 (*p* = 0.0467) for GB

Correlations of preoperative PhA with %EWL were significant up to 24 months. Regarding total weight loss (%TWL), correlations were significant up to 3 months following SG and up to 24 months after GB (see Table 2). The correlation between preoperative PhA and %TWL 12 months after SG and GB is visualized in Fig. 1.

**Table 2:** Correlation of phase angle with weight loss parameters following sleeve gastrectomy (SG) and gastric bypass (GB) over a period of 24 months

		6 weeks	3 months	6 months	12 months	24 months
TWL (%)	SG	*0.31048*	*0.31444*	0.11663	0.19988	0.22994
		*0.0266*	*0.0218*	0.3666	0.1360	0.1120
	GB	0.14402	0.18894	*0.23956*	*0.19184*	*0.23203*
		0.1528	0.0713	*0.0071*	*0.0467*	*0.0371*
EWL (%)	SG	0.44133	*0.44036*	0.21897	*0.33819*	*0.30834*
		*0.0012*	*0.0010*	0.0873	*0.0101*	*0.0311*
	GB	0.15517	0.16286	*0.23313*	*0.22317*	*0.22806*
		0.1232	0.1209	*0.0089*	*0.0203*	*0.0406*

The *r* value is presented in the top line and the *p* value in the bottom line. Significant correlations are printed in italic characters

*EWL* excess weight loss, *TWL* total weight loss

There was a significantly negative correlation of preoperative PhA with the percentage of body fat (SG: *r* = − 0.55349, *p* < 0.0001; GB: *r* = − 0.24313, *p* = 0.0287) and BCM (SG: *r* = 0.32814, *p* = 0.0213; GB: *r* = 0.36356, *p* = 0.0008) up to 24 months. In the GB group, there was a significant correlation with LBM up to 12 months after surgery (*r* = 0.20933, *p* = 0.0297).

##### Correlation of Handgrip Strength with Weight Loss and Postoperative Body Composition

There was no significant correlation of preoperative handgrip strength of the dominant hand with %TWL at any time point examined postoperatively after SG and GB (the coefficients after 12 months were *r* = 0.01564, *p* = 0.9081 for SG and *r* = 0.11411, *p* = 0.2396 for GB).

Preoperative handgrip strength and the percentage of body fat, however, showed a significantly negative correlation at all time points examined up to 24 months following SG and GB. Our results showed a significantly positive correlation with LBM and BCM at all time points up to 24 months in both groups (see Table 3).

**Table 3:** Correlation of handgrip strength with parameters of body composition in bioelectrical impedance analysis following sleeve gastrectomy (SG) and gastric bypass (GB) over a period of 24 months

		6 weeks	3 months	6 months	12 months	24 months
LBM (kg)	SG	*0.73937*	*0.61423*	*0.62014*	*0.64406*	*0.57481*
		*< 0.0001*	*< 0.0001*	*< 0.0001*	*< 0.0001*	*< 0.0001*
	GB	*0.56527*	*0.45621*	*0.48745*	*0.36033*	*0.46737*
		*< 0.0001*	*< 0.0001*	*< 0.0001*	*0.0001*	*< 0.0001*
Body fat (%)	SG	*− 0.58913*	*− 0.51162*	*− 0.47704*	*− 0.47490*	*− 0.39042*
		*< 0.0001*	*< 0.0001*	*< 0.0001*	*0.0002*	*0.0055*
	GB	*− 0.39668*	*− 0.25153*	*− 0.32455*	*− 0.28274*	*− 0.27038*
		*< 0.0001*	*0.0156*	*0.0002*	*0.0030*	*0.0146*
BCM (%)	SG	*0.76301*	*0.64853*	*0.62931*	*0.69658*	*0.68000*
		*< 0.0001*	*< 0.0001*	*< 0.0001*	*< 0.0001*	*< 0.0001*
	GB	*0.54882*	*0.47661*	*0.44608*	*0.33036*	*0.48965*
		*< 0.0001*	*< 0.0001*	*< 0.0001*	*0.0005*	*< 0.0001*

The *r* value is presented in the top line and the *p* value in the bottom line. Significant correlations are printed in italic characters

*BCM* body cell mass, *LBM* lean body mass

### Logistic Regression Analysis

#### PhA as a Predictive Marker

**Fig. 2:**
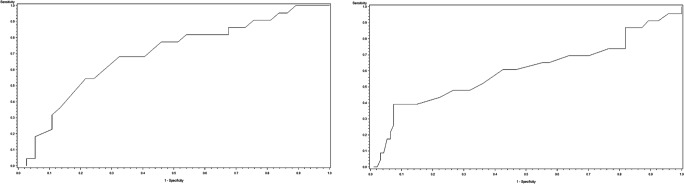
ROC analysis for the phase angle as a predictor for a surgical response of less than 50% EWL 12 months after **a** sleeve gastrectomy (AUC = 0.697, *p* = 0.0300) and after **b** gastric bypass (AUC = 0.600, *p* = 0.1425)

Of the 59 patients who underwent SG and were included in a logistic regression analysis, 22 patients showed response of less than 50% EWL 1 year after SG. A univariable logistic regression analysis revealed an area under the curve (AUC) of 0.697 (*p* = 0.0300), representing a fair model for the prediction of a less pronounced weight loss after SG. The optimum cutoff point in the curve was a preoperative PhA of 6.0°, which delivered a sensitivity of 68% and a specificity of 68% for predicting a response of less than 50% EWL after SG. The corresponding ROC curve is displayed in Fig. 2a.

After GB, 23 of 117 patients failed to show an EWL of more than 50% 1 year after surgery. Univariable logistic regression analysis for GB revealed an AUC of 0.600 (*p* = 0.1425), representing a modest model for the prediction of a less pronounced weight reduction after GB. The optimum cutoff point in the curve was a preoperative PhA of 6.0°, which delivered a sensitivity of 61% and a specificity of 57% for predicting a response of less than 50% EWL after GB. The corresponding ROC curve is displayed in Fig. 2b.

#### Handgrip Strength as a Predictive Marker

The preoperative handgrip strength did not serve as a good model for the prediction of a less pronounced weight loss after bariatric surgery (SG: AUC = 0.516 with *p* = 0.7163; GB: AUC = 0.515 with *p* = 0.3610).

#### Lean Body Mass as a Predictive Marker

Preoperative LBM cannot be considered a useful marker. For both types of surgery, the impact on a response of less than 50% EWL is not significant (SG: AUC = 0.585 with *p* = 0.3994; GB: AUC = 0.529 with *p* = 0.0943).

#### Factors Influencing Insufficient Weight Loss

In multiple analyses, preoperative phase angle, gender, preoperative size, and body weight were revealed as significant factors predicting a less pronounced weight loss after bariatric surgery (Table 4).

**Table 4:** Variables significantly affecting a response of less than 50% excess weight loss after bariatric surgery

Variable	OR	*p*
Preoperative PhA	0.650	0.0796
Gender (male vs. female)	3.673	0.0498
Size	0.873	0.0005
Preoperative body weight	1.046	< 0.0001

No significance: BMI, handgrip strength, lean body mass, recurrent surgery, type of surgery

*PhA* phase angle

## Discussion

We were able to demonstrate for the first time in a large study population that PhA and handgrip strength are predictive factors for the effectiveness and quality of postoperative weight loss after bariatric surgery.

In spite of the preference to use %TWL to assess the bariatric outcome [4, 7], most studies still use %EWL to define a less pronounced weight loss. A %EWL of 50–80% is expected 1–3 years after surgery [2, 8]. In accordance with previous studies, we defined a %EWL less than 50% 1 year after bariatric surgery as being an inadequate loss of weight [5, 9].

In Table 5 an overview of predictors of postbariatric outcome identified by previous studies is presented. Our results show a significant predictive ability for the nonmodifiable parameters gender and size as well as the potentially modifiable factors preoperative PhA and preoperative body weight. Preoperative body weight in kilograms seems to be the most important independent risk factor, which was also shown in a meta-analysis published by Livhits et al. [10].

**Table 5:** Overview of predictors of postbariatric weight loss in different studies

Authors, year	Type of study	Number	Gender (F/M)	Mean age (years)	Type of surgery (GB/SG/other)	Positive predictors	Negative predictors	FU (months)
Vassilev et al., 2017 [5]	Prospective	173	122/51	-	127/46/0	PhA	-	12
Otto et al., 2014 [6]	Prospective	25	16/9	36.8 F 46.7 M	19/6/0	Handgrip strength	-	4
Goldenshluger et al., 2017 [15]	Retrospective	201	121/80	39.9	0/201/0	Increased physical activity	Age, high basal BMI, polypharmacy	36
Al-Khyatt et al., 2017 [9]	Prospective	227	164/63	48.6	227/0/0	Preoperative EWL	Age, T2DM, OSA, TtS, high initial BMI	12
Perrone et al., 2016 [16]	Retrospective	304	210/94	41.8 SG 43.8 GB	142/162/0	Gender (male)—sleeve op	Age	60–96
Limbach et al., 2014 [17]	Retrospective	415	316/99	47.38	415/0/0	Preoperative WL, Caucasian race	Age, high initial BMI	12
Ortega et al., 2012 [18]	Retrospective	407	309/98	44	307/100/0	-	Age, fasting glucose, HbA1c	12

*FU*, follow-up, *GB* gastric bypass, *SG* sleeve gastrectomy

Bariatric surgery is associated with a substantial decrease in LBM and muscle strength, leading to a reduction in metabolic rate [4]. Therefore, factors representing the functionality and amount of the initial muscle mass, such as preoperative PhA and handgrip strength, have shown an encouraging ability to predict the bariatric outcome [5, 6]. Our study shows a significant correlation between both parameters. However, in spite of the evidence of a correlation, neither preoperative handgrip strength alone nor the combination of handgrip strength and PhA was suitable as a predictor for postoperative weight loss.

The results of the present study are in accordance with the results of the study by Otto et al. [6], who showed a correlation between preoperative handgrip strength and postoperative body composition in a retrospective case series including 25 patients followed for 18 weeks postoperatively. Our results show significant correlations of handgrip strength with BCM, LBM, and the percentage of body fat up to 24 months after surgery.

Vassilev et al. [5] showed a positive correlation between the PhA and %EWL up to 12 months after surgery in a retrospective case series of 173 patients. In our slightly larger patient cohort (*n* = 198), the preoperative PhA also correlated with %TWL up to 3 months after SG and up to 2 years after GB. We determined a higher preoperative PhA cutoff value for predicting a marked postoperative weight loss. As far as the difference in cutoff values for PhA is concerned, the definition of cutoff values is very sensitive. However, a cutoff of 6.0° seems to be more realistic than 3.9° because of the distribution of PhA values (median 6.1°, range 4.0–9.7°). As is well known, there is a decrease in the amount of musculature after bariatric surgery, and consequently, the PhA decreased to 5.10° following SG and 5.18° following GB after 24 months postoperatively (Supplementary Table 2).

The association between preoperative muscle mass and postoperative outcome has been investigated in several different studies. A low value for muscle mass derived from preoperative imaging, for instance, is related to a significantly higher rate of major postoperative complications in patients with Crohn’s disease [11] and of overall complications after colon resection [12]. Previous research showed that the decrease in fat mass is accompanied by a reduction of LBM [13, 14]. Postoperative oxygen metabolization in muscles is reduced after SG, being correlated with the loss of metabolically active LBM [14]. The aim of bariatric surgery, however, should be to preserve muscle mass postoperatively in favor of a greater reduction of fat mass. Quantitative muscle mass is represented by LBM in BIA. The preservation of LBM is essential for sustained weight loss because muscle tissue has a higher metabolic rate than adipose tissue. Handgrip strength and PhA are qualitative measures of muscle mass. Interestingly, even though PhA in theory reflects LBM, LBM is not a suitable preoperative surrogate variable for the outcome of weight loss, whereas the independent impact of the preoperative PhA was confirmed by our multiple analyses.

## Limitations of the Study

The strength of this study’s results is limited by all the well-known bias risks of cohort studies, stemming, for example, from a nonrandomized design or loss to follow-up. However, since no intervention was performed, a randomized design is not necessary for this type of question. Furthermore, as represented by the AUC, the model using the PhA as a predictor for less pronounced weight loss after bariatric surgery has limited validity, and the sensitivity of the PhA is comparably low (68% for SG and 61% for GB).

## Conclusion

To our knowledge, this is the largest study to report an investigation of the postoperative changes in body composition and the role of preoperative handgrip strength and PhA as predictors of postoperative success following bariatric surgery. We showed again that the quality of preoperative body composition correlates with postbariatric outcome. We confirmed handgrip strength to be suitable as a predictor of postoperative body composition not only in the short term but also in a long-term assessment of BIA. The PhA has the potential to predict the effectiveness of postoperative weight loss. Since the outcome of bariatric surgery could potentially be affected positively by improving the preoperative body composition, further studies are warranted to analyze the extent to which some preconditioning, such as preoperative training and nutrition, might influence these parameters in order to optimize postoperative weight loss after bariatric surgery.

## Electronic Supplementary Material

ESM 1 (DOCX 22 kb)
