# Supplementary material for: Handgrip Strength and Phase Angle Predict Outcome After Bariatric Surgery
Source: Obes Surg. 2020 Aug 15;31(1):200–6. doi: 10.1007/s11695-020-04869-7 (PMC7808965; doi:10.1007/s11695-020-04869-7)
Supplement: Supplementary file 1 — (DOCX 22 kb) [file 11695_2020_4869_MOESM1_ESM.docx]

**Supplementary Material**

**Number of patient exclusion and reasons for non-enrolment**

Between January 2013 and December 2016, a total of 254 laparoscopic SG and GB were performed in our center. Fifty-six patients were excluded for the following reasons: missing preoperative static muscle strength values (24 patients), lacking or insufficient preoperative or postoperative BIA measurements (6 and 20 patients, respectively), relevant cointerventions (5 patients), and postoperative death (1 patient). Thus, a total of 198 patients were included in this prospective cohort analysis.

**Follow-up periods**

We assigned follow-up dates for postoperative assessment to certain follow-up periods. The first date for postoperative assessment, 6 weeks after bariatric surgery, included patients who turned up for follow-up assessment within the first 10 weeks after surgery. The second date, after 3 months, included patients followed up in the period 10-18 weeks after surgery. The third date, after 6 months, included patients whose follow-up appointments were in the period from 18 weeks to 9 months after surgery. The period for the fourth date, after 12 months, was 9-15 months; for the fifth date, after 24 months, 17-28 months; and for the sixth date, after 36 months, 28-40 months following bariatric surgery.

**Completeness of follow-up**

At 6 weeks after surgery, 151 patients (76.3%) had complete follow-up data. One hundred forty-five patients (73.2%) were observed 3 months after surgery. A total of 187 patients (94.4%) were followed up for 6 months, 165 (83.3%) for 12, 130 (65.7%) for 24, and 66 (33.3%) for 36 months after surgery. Because of the amount of data missing for 36 months after surgery, we focused on 24 months in order to evaluate treatment success.

**Postoperative Changes**

The postoperative course of weight loss after laparoscopic sleeve gastrectomy (SG) and laparoscopic Roux-Y gastric bypass (GB) is presented in Supplementary Table 1. ANOVAs for repeated measurements led to p < 0.0001 for each parameter and for each treatment group. The maximum %EWL was almost already reached 12 months after surgery. The mean %EWL 24 months after surgery was higher for GB (66.58 ± 21.91%) than for SG (53.71 ±16.78%) without reaching significance when adjusted for baseline body weight (p = 0.1028). The mean %TWL 24 months after surgery was similar in both groups (p = 0.2705, adjusted for baseline body weight). We have confirmed here the lack of a relevant difference between the outcomes for SG and GB regarding effective weight loss. In our study, the mean overall %EWL was 61.73% two years after surgery. Our result tends to be lower than that of other studies since the baseline body measurements in this study were taken after a two-week period on a liquid low-calorie diet.

The postoperative course of mean phase angle, body mass index, and parameters of bioelectrical impedance analysis (BIA) is presented in Supplementary Table 2. ANOVAs for repeated measurements led to p < 0.0001 for each parameter and for each treatment group.

The mean BMI continuously decreased to (39.64 ± 7.21) kg/m^2^ and (32.75 ± 5.28) kg/m^2^, respectively, after 12 months before reaching a plateau (p = 0.3663, adjusted for baseline BMI).

After the operation a continuous reduction of the mean PhA to (5.10° ±0.91) after SG and (5.18° ±0.89) after GB was recorded (p = 0.2608, adjusted for baseline PhA).

Mean LBM decreased to (71.07 ±15.77) kg after SG and (59.15 ±9.68) kg 24 months after GB (p = 0.8983, adjusted for baseline LBM). The mean preoperative relative amount of body fat decreased to (36.95 ± 9.71) % and (33.81 ± 8.01) %, respectively, 24 months after surgery (p = 0.0111, adjusted for baseline parameter), and the BCM decreased to (33.73 ±9.16) kg and (28.15 ±6.25) kg 24 months after surgery (p = 0.5029, adjusted for baseline BCM).

**Supplemantary Table 1** Course of weight loss expressed as percentage of mean total weight loss (%TWL), excess weight loss (%EWL), and excess BMI loss (%EBL) over a period of 24 months following sleeve gastrectomy (SG) and Roux-en-Y gastric bypass (GB)

|  |  | 6 weeks | 3 months | 6 months | 12 months | 24 months |
| --- | --- | --- | --- | --- | --- | --- |
| SG | TWL % | 11.46 (3.25) | 15.57 (4.05) | 21.82 (6.05) | 27.20 (7.31) | 28.14 (8.77) |
|  | EWL % | 22.41 (7.16) | 30.14 (9.41) | 41.87 (13.41) | 52.35 (15.61) | 53.71 (16.78) |
|  | EBL % | 22.41 (7.16) | 30.14 (9.41) | 41.87 (13.41) | 52.35 (15.61) | 53.71 (16.78) |
|  |  |  |  |  |  |  |
| GB | TWL % | 10.63 (2.99) | 15.96 (3.66) | 22.26 (5.57) | 28.61 (7.30) | 29.06 (9.01) |
|  | EWL % | 24.37 (8.68) | 36.31 (10.51) | 50.72 (15.01) | 65.42 (19.54) | 66.58 (21.91) |
|  | EBL % | 24.37 (8.68) | 36.31 (10.51) | 50.72 (15.01) | 65.42 (19.54) | 66.58 (21.91) |

Data is presented as mean and SD in parenthesis. P < 0.0001 (repeated measures ANOVA) for each parameter and each treatment group.

**Supplementary Table 2** Course of mean phase angle, body mass index, and bioelectrical impedance analysis following sleeve gastrectomy (SG) and gastric bypass (GB) over a period of 24 months

|  |  | 0  Preop. | 6  weeks | 3  months | 6  months | 12  months | 24  months |
| --- | --- | --- | --- | --- | --- | --- | --- |
|  | n SG/BP | 68/130 | 51/100 | 53/92 | 62/125 | 57/108 | 49/81 |
| PhA (°) | SG | 6.30  (1.09) | 5.40  (0.81) | 5.40  (0.73) | 5.17  (0.87) | 5.16  (0.77) | 5.10  (0.91) |
|  | GB | 6.11  (0.77) | 5.28  (0.74) | 5.12  (0.70) | 5.24  (0.76) | 5.08  (0.91) | 5.18  (0.88) |
| BMI (kg/m^2^) | SG | 54.28 (8.22) | 47.27 (6.89) | 45.94 (7.68) | 42.76 (7.14) | 39.64 (7.21) | 38.86 (6.93) |
|  | GB | 45.93 (5.24) | 41.10 (5.20) | 38.62 (4.74) | 35.70 (4.96) | 32.75 (5.28) | 32.19 (5.21) |
| LBM (kg) | SG | 82.15 (20.08) | 76.66 (17.08) | 74.57 (16.89) | 72.54 (15.70) | 71.23 (15.59) | 71.07 (15.77) |
|  | GB | 67.20 (12.07) | 63.21 (11.60) | 62.44 (10.75) | 61.34 (9.96) | 59.40  (9.59) | 59.15 (9.59) |
| Body fat (%) | SG | 48.33 (7.97) | 45.14 (8.23) | 44.24 (8.82) | 41.69 (7.74) | 38.34 (7.74) | 36.95 (9.71) |
|  | GB | 47.64 (6.67) | 44.70 (7.18) | 42.06 (6.89) | 38.21 (7.60) | 34.32 (8.27) | 33.81 (8.01) |
| BCM (%) | SG | 43.78 (12.63) | 37.30 (9.58) | 36.29 (8.82) | 34.38 (9.11) | 33.81 (8.73) | 33.73 (9.16) |
|  | GB | 35.23 (7.57) | 30.43 (6.90) | 29.50 (6.22) | 29.33 (5.73) | 27.79 (5.99) | 28.15 (6.45) |

Data is presented as mean and SD in parenthesis. P < 0.0001 (repeated measures ANOVA) for each parameter and each treatment group.

Legend: BCM, body cell mass; BMI, body mass index; LBM, lean body mass; PhA, phase angle.
